# Supplementary material for: Fine mapping of MHC region in lung cancer highlights independent susceptibility loci by ethnicity
Source: Nat Commun. 2018 Sep 25;9:3927. doi: 10.1038/s41467-018-05890-2 (PMC6156406; doi:10.1038/s41467-018-05890-2)
Supplement: Supplementary file 1 — Supplementary Information [file 41467_2018_5890_MOESM1_ESM.pdf]

Supplementary Information

Fine mapping of MHC region in lung cancer highlights independent susceptibility loci by ethnicity

Ferreiro-Iglesias *et al.*

Acronym	Study Name	Institute	PI	Country	Study design	Study period	EU Cases*	EU Controls*	AS Cases*	AS Controls*	Total*	Ref (PMID)
ATBC	The Alpha-Tocopherol, Beta-Carotene Cancer Prevention	NCI	D. Albanes	Finland	Cohort	1985-1993	1,017	666	0	0	1,683	8205268
CANADA	Canadian screening study	UHN, BCCA	S.Lam/M-S Tsao	Canada	Screening cohort	2004-2011, 2008-2013	215	443	3	10	671	17065637
CAPUA	Cáncer de Pulmón en Asturias	U. Oviedo	A. Tardon	Spain	Hosp.CC	2002-2012	717	686	0	0	1,403	23013535
CARET	The Carotene and Retinol Efficacy Trial	FHCRC	J. Doherty, C. Chen	USA	Cohort	Recruitment 1985-1996.	550	520	3	4	1,077	15572756
COPENHAGEN	Copenhagen lung cancer study	U.Copenhagen	S. E. Bojesen	Denmark	Hosp.CC	2004(controls)2012-2013 (cases)	583	1,410	7	5	2,005	22441734
EAGLE	Environment and Genetics in Lung Cancer Study Etiology	NCI	M.T. Landi	Italy	Pop.CC	2002-2005	1,803	1,750	0	0	3,553	18538025
LLP	Liverpool Lung Cancer Project	U. Liverpool	J.K. Field	UK	Cohort	1999-2007, 1999-2011	429	466	0	6	901	25873368
GERMANY	German lung cancer study	HMGU, DKFZ	H. Bickeböller, A. Risch	Germany	Mixed CC	1998-2013	818	221	3	3	1,045	18483334
HLCS	Harvard Lung Cancer Study	Harvard/Mass General Hospital	D. Christiani	USA	Hosp. CC	1992-2004	2,685	702	53	8	3,448	25873368
EPIC	European Prospective Investigation into Cancer and Nutrition	IARC	M. Johansson	Europe	Cohort	1992-1999	1,195	1,220	2	1	2,418	12639222
NICCC-LCA	Clalit National Israeli Cancer Control Center- lung cancer study	Carmel Medical Center	G. Rennert	Israel	Pop.CC	2008-ongoing	662	510	0	0	1172	25924736
LCRI-DOD	LCRI-DOD	Markey Cancer Center	S. Arnold	USA	Pop.CC	2012-2014	93	131	0	0	224	
MDACC	MD Anderson Cancer Center Study	MDACC	X. Wu	USA	Hosp. CC	1994-present	972	954	1	1	1,928	8429286
MDCS	The Malmö Diet and Cancer Study	Lund University	J. Manjer	Sweden	Cohort	1991-1996	158	168	0	0	326	11916347
MEC	Multiethnic Cohort Study	U of Hawaii, USC	L. Le Marchand	USA	Cohort	Recruitment 1993-1996	243	240	370	404	1,257	15229477
NELCS	New England Lung Cancer Study	Dartmouth College of Medicine	A. Andrew	USA	Pop. CC	2005-2007	172	171	1	0	344	20049123
NIJMEGEN	The Nijmegen Lung Cancer Study	Radboud University Medical Centre	L. A. Kiemeney, E. van der Heijden	The Netherlands	Pop. CC	2002-2008	420	443	3	1	867	Control:17568781; Case:20418888, 25924736
NORWAY	Norway Lung Cancer Study	NIOH	A. Haugen	Norway	Pop. CC	1986-2005	320	417	0	0	737	18258609
NSHDS	Northern Sweden Health and Disease Study	Umeå University	M. Johansson	Sweden	Cohort	1985-present	237	236	0	0	473	14660243
PLCO	The Prostate, Lung, Colorectal and Ovarian Cancer Screening Trial	NCI	N. Caporaso	USA	Cohort	1992-2001	1353	890	18	15	2,276	17065637
RESOLUCENT	ReSoLucent	U. Sheffield	Woll	UK	Mixed CC	2005-2014	576	297	7	0	880	8637137
L2	The IARC L2 study	IARC	P. Brennan, G. Scelo	CE Europe	Hosp/Pop CC	2005-2013	1,084	1,072	0	0	2,156	
TAMPA	Tampa Lung Cancer Study	WSU	P. Lazarus	USA	Hosp. CC	1999-2003	101	146	4	2	253	
TLC	Total Lung Cancer: Molecular Epidemiology of Lung Cancer Survival	Moffitt Cancer Center, Tampa, FL	M.B. Schabath	USA	Case only	2012-present	427	0	3	0	430	23839018
MSH-PMH	Mount Sinai Hospital-Princess Margaret Hospital Study	MSH, PMH	R.J. Hung, G. Liu	Canada	Hosp. CC	2008-2012	1,371	953	0	0	2,324	24880342, 24947688
BioVU	Vanderbilt 2	Vanderbilt	M. Aldrich	USA	Hosp. CC	2007-present	723	727	2	2	1,454	
SCS	Shanghai Cohort Study	U. Pittsburgh	J.M. Yuan	China	Cohort	1986-1989 baseline	0	0	170	317	487	8637137
SCHS	Singapore Chinese Health Study	U. Pittsburgh	J.M. Yuan	Singapore	Cohort	1993-1998	0	0	408	412	820	14504200
NJLCS	Nanjing Lung Cancer Study	Nanjing Medical University	H.Shen	China	Community CC	2004-2012	0	0	978	0	978	
Seoul	Bundang lung cancer study	Seoul National University	Y-C. Hong	S. Korea	Hosp.CC	2008-2010	0	0	288	465	753	
**Total**							**18,924**	**15,439**	**2,324**	**1,656**	**38,343**	

Hosp. Hospital-based; Pop., Population-based; CC, case-control study; EU, European; AS, Asian; CE, Central and Eastern Europe
*numbers of cases and controls after quality control filters.

Supplementary Table 1: Detailed characteristics of each of the Oncoarray studies included for the analyses.

Locus	HLA allele	Frequency		Unconditional analysis [a]	
		Controls[c]	Cases[c]	OR (95%CI)	P value
AH 8.1	*A1-B8-DR3-DQ2*[c]	-	-	-	-
HLA-A	*01:01*	0.01	0.02	1.71 (1.06-2.78)	0.03
HLA-C	*07:01*	0.02	0.01	0.89 (0.49-1.62)	0.71
HLA-B	*08:01*	0.002	0.004	2.29 (0.64-8.21)	0.20
HLA-DRB1	*03:01*	0.04	0.04	0.90 (0.63-1.26)	0.53
HLA-DQA1	*05:01*	0.15	0.15	0.92 (0.76-1.11)	0.38
HLA-DQB1	*02:01*	0.08	0.09	1.05 (0.83-1.34)	0.67
HLA-DQB1	*06 global*[b]	0.22	0.22	1.06 (0.90-1.25)	0.47
	06:01	0.09	0.10	1.13 (0.89-1.43)	0.31
	06:02	0.067	0.074	1.17 (0.90-1.51)	0.24
	06:03	0.02	0.01	0.70 (0.37-1.29)	0.25
	06:04	0.02	0.01	0.75 (0.43-1.31)	0.32
	06:09	0.02	0.02	0.97 (0.60-1.57)	0.90
	*06:10**	0.0005	0.0002	0.08 (0-898106.59)	0.76

AH 8.1, ancestral haplotype 8.1; HLA, human leucocyte antigen; OR, odds ratio; 95%CI, confidence interval

[a] Obtained from multivariate logistic regression assuming an additive genetic model with sex and principal components as covariates

[b] Classical two-digit allele corresponding to the four digit alleles found (*0601,*0602,*0603,*0604,*0609,*0610)

[c] Number of samples included in the analysis: 641 cases and 1,656 controls

* not passed imputation QC criteria in Asians

The study-wide significant threshold was P=6 x 10^{-6} (Bonferroni correction)

[c] AH 8.1 is absent in the Asian samples

Supplementary Table 2: Top associations of the HLA alleles with lung squamous cell carcinoma of European ancestry shown for Asians Oncoarray.

Locus	Variant	Frequency		Multivariate analysis[a]	
		Controls[b]	Cases[b]	OR (95%CI)	P value
HLA-DQB1	*04:01**	0.0003	0.0002	0.55 (0.07-4.26)	0.57
HLA-DRB1	*07:01*	0.13	0.13	0.95 (0.89-1.01)	0.09
HLA_A (intronic)	rs2256919	0.42	0.42	0.99 (0.95-1.03)	0.68

HLA, human leucocyte antigen; OR, odds ratio; 95%CI, confidence interval
[a] Obtained from multivariate unconditional logistic regression assuming an additive genetic model with sex and principal components as covariates
[b] Number of samples included in the analysis: 7,088 cases and 15,439 controls

The study-wide significant threshold was P=6 x 10^{-6} (Bonferroni correction)

* not passed imputation QC criteria in Asians

Supplementary Table 3: Top associations of the HLA alleles with adenocarcinoma of Asian ancestry (Oncoarray) shown for Europeans.

Locus	HLA variant	Unconditional analysis[a]	BIC	
		P value	Value	BIC difference[i]
AH 8.1	*A1-B8-DR3-DQ2*	4.78x10^{-8}	21624	+14
HLA-A	*01:01*	9.36 x10^{-7}	21633	+23
HLA-C	*07:01*	5.53 x10^{-7}	21628	+18
HLA-B	*08:01*	9.01 x10^{-9}	21627	+17
	**163(Thr)[c]**	**1.30** x10^{-11}	**21610**	**0**
	9(Asp)[d]	6.86 x10^{-9}	21626	+16
	156(Asp)[e]	2.76 x10^{-8}	21632	+22
HLA-DRB1	03:01	6.38 x10^{-10}	21618	**+8**
	71(Lys)[f]	1.38 x10^{-8}	21630	+20
	74(Arg)[g]	7.27 x10^{-10}	21617	**+7**
	26(Tyr)[h]	7.39 x10^{-9}	21621	+11
HLA-DQA1	05:01	1.33 x10^{-9}	21616	**+6**
HLA-DQB1	02:01	4.45 x10^{-10}	21615	**+5**
HLA-DQB1	06 global[b]	3.05 x10^{-8}	21617	**+7**
	125 (Gly)	3.05 x10^{-8}	21617	**+7**
	87(Phe)	3.60 x10^{-6}	21624	+14
	06:01	0.3	21652	+42
	06:02	0.007	21644	+34
	06:03	5.06 x10^{-4}	21641	+31
	06:04	0.03	21644	+34
	06:09	0.1	21650	+40

AH 8.1, ancestral haplotype 8.1; HLA, human leucocyte antigen; BIC, bayesian information criterion, SCC squamous cell carcinoma

[i] models having their BIC within: +1–2 of the minimum have substantial support; + 4–7 of the minimum have considerably less support; BIC >10 above the minimum have either essentially no support

[a] Obtained from multivariate unconditional logistic regression assuming an additive genetic model with sex and principal components as covariates

[b] Classical two-digit allele corresponding to the four digit alleles found (*06:01,*06:02,*06:03,*06:04,*06:09)

The study-wide significant threshold was P=6 x 10^{-6} (Bonferroni correction)

[c,d,e,f,g,h] Alleles where these amino acids are part of the sequence: 163(Thr) in*0801 and also *4102, *4101, *3701, *1801; *1402; *1401; 9(Asp) only *0801; 156(Asp) in *0801 and also *4102, *4101, *4402, *4501; 71(lys) in *0301 and also *0401, *1303; 74(Arg) only in *0301; 26(Tyr) in *0301 and also *0901

Supplementary Table 4: Unconditional model comparison using BIC in SCC of European ancestry

Locus	Variant	Frequency		Unconditional analysis[a]		BIC	
		Controls[b]	Cases[b]	OR (95%CI)	P value	Value	BIC difference[i]
HLA-DQB1	*04:01*	0.06	0.09	1.67 (1.35-2.05)	1.59×10^{-6}	3860	+5
	23(Leu)	0.06	0.09	1.66 (1.35-2.05)	1.70×10^{-6}	3860	+5
HLA-DRB1	*07:01*	0.05	0.09	1.62 (1.31-2.01)	5.48×10^{-6}	3857	+2
	104(Ala)	0.36	0.43	1.34 (1.20-1.50)	1.96×10^{-7}	3855	0
	98(Glu)	0.36	0.43	1.34 (1.20-1.50)	1.97×10^{-7}	3855	0
HLA_A (intronic)	rs2256919	0.44	0.38	0.75 (0.67-0.83)	1.75×10^{-7}	3855	0

HLA, human leucocyte antigen; OR, odds ratio; 95%CI, confidence interval

[i] models having their BIC within: +1–2 of the minimum have substantial support; + 4–7 of the minimum have considerably less support; BIC >10 above the minimum have either essentially no support, fail to explain some substantial structural variation in the data

[a]Obtained from multivariate unconditional logistic regression assuming an additive genetic model with sex and principal components as covariates

[b] Number of samples included in the analysis: 1,192 cases and 1,656 controls

[i] models having their BIC within: +1–2 of the minimum have substantial support; + 4–7 of the minimum have considerably less support; BIC >10 above the minimum have either essentially no support

Supplementary Table 5: Unconditional models and comparison with the minimum BIC value in AD of Asian ancestry (Oncoarray)

Model	Locus	OR (95%CI)	P value	BIC value	BIC dif[i]
A	DQB1*0401 + DRB1*0701 + rs2256919			3839	0
	DQB1*0401	1.73 (1.41-2.14)	2.85×10^{-7}		
	DRB1*0701	1.63 (1.32-2.03)	5.34×10^{-6}		
	rs2256919	0.76 (0.68-0.85)	8.92×10^{-7}		
B	DRB1-Ala104 + DQB1-leu23 + rs2256919			3855	+16
	DQB1-leu23	1.46 (1.17-1.82)	1.3×10^{-6}		
	DRB1-Ala104	1.22 (1.08-1.37)	2.0×10^{-6}		
	rs2256919	0.75 (0.68-0.84)	8.39×10^{-7}		

BIC, Bayesian information criterion; HLA, human leukocyte antigen; AD, adenocarcinoma

[a]Obtained from multivariate unconditional logistic regression assuming an additive genetic model with sex and principal components as covariates

[i] Models having their BIC within: +1–2 of the minimum have substantial support; + 4–7 of the minimum have considerably less support; BIC >10 above the minimum have either essentially no support, fail to explain some substantial structural variation in the data

The study-wide significant threshold was P=6 x 10^{-6} (Bonferroni correction)

Supplementary Table 6: HLA alleles and amino acids contained in the best models obtained for AD of Asian ancestry (Oncoarray) judged by the BIC

	Asian replication	
	Case no.(%)	Control no.(%)
**Lan et al. passed QC[a]**	4796	3741
Age		
≤50	976 (20.4)	851 (22.7)
>50	3820 (79.6)	2890 (77.3)
Missing	0 (0)	0 (0)
Sex		
Male	0 (0)	0 (0)
Female	4796 (100)	3741 (100)
Missing	0 (0)	0 (0)
Smoking status		
Never	4796 (100)	3741 (100)
Ever	0 (0)	0 (0)
Missing	0 (0)	0 (0)
Histology		
Adenocarcinoma	3469 (72.3)	-
Squamous cell carcinoma	660 (13.7)	-
Others	667 (14.0)	-

Supplementary Table 7. Demographic characteristics of the replication study after quality control filters

Locus	HLA variant	Frequency		Unconditional analysis[a]	
		Controls	Cases	OR (95%CI)	P value
AH 8.1	*A1-B8-DR3-DQ2*	0.06	0.07	1.28 (1.16-1.41)	4.85E-07
HLA-A	*01:01*	0.15	0.17	1.17 (1.09-1.25)	8.30E-06
HLA-C	*07:01*	0.15	0.17	1.18 (1.1-1.26)	1.08E-06
HLA-B	*08:01*	0.10	0.12	1.25 (1.15-1.35)	4.03E-08
	163(Thr)[c]	0.28	0.31	1.2 (1.14-1.27)	3.02E-11
	9(Asp)[d]	0.10	0.12	1.25 (1.15-1.35)	3.40E-08
	156(Asp)[e]	0.21	0.24	1.18 (1.11-1.25)	4.96E-08
HLA-DRB1	*03:01*	0.11	0.14	1.25 (1.16-1.34)	5.84E-09
	71(Lys)[f]	0.2	0.23	1.19 (1.12-1.27)	1.68E-08
	74(Arg)[g]	0.11	0.14	1.24 (1.16-1.34)	6.64E-09
	26(Tyr)[h]	0.12	0.15	1.22 (1.14-1.31)	4.39E-08
HLA-DQA1	*05:01*	0.2	0.29	1.17 (1.11-1.24)	2.40E-08
HLA-DQB1	*02:01*	0.11	0.14	1.24 (1.15-1.34)	7.19E-09
HLA-DQB1	*06 global*[b]	0.24	0.21	0.84 (0.79-0.89)	1.06E-08
	125 (Gly)	0.24	0.21	0.84 (0.79-0.89)	1.07E-08
	87(Phe)	0.20	0.17	0.85 (0.8-0.91)	1.37E-06
	06:01	0.01	0.01	0.85 (0.65-1.11)	2.42E-01
	06:02	0.12	0.10	0.9 (0.83-0.98)	0.012
	06:03	0.07	0.06	0.81 (0.73-0.9)	7.37E-05
	06:04	0.04	0.03	0.85 (0.74-0.98)	0.03
	06:09	0.009	0.007	0.81 (0.61-1.08)	0.14

AH 8.1, ancestral haplotype 8.1; HLA, human leucocyte antigen; OR, odds ratio; 95%CI, confidence interval

[a] Obtained from multivariate unconditional logistic regression assuming an additive genetic model with sex, smoking status and principal components as covariates

[b] Classical two-digit allele accounting for the four digit alleles found (*0601,*0602,*0603,*0604,*0609) which are sharing the amino acids 125(Gly) and 87 (Phe)

The study-wide significant threshold was P=6 x 10^{-6} (Bonferroni correction)

[c,d,e,f,g,h] Alleles where these amino acids are part of the sequence: 163(Thr) in*0801 and also *4102, *4101, *3701, *1801; *1402; *1401; 9(Asp) only *0801; 156(Asp) in *0801 and also *4102, *4101, *4402, *4501; 71(lys) in *0301 and also *0401, *1303; 74(Arg) only in *0301; 26(Tyr) in *0301 and also *0901

Supplementary Table 8: Top associations of the HLA alleles with squamous cell carcinoma of European ancestry including **smoking** as a covariate on top of sex and principal components used in the ordinary analyses.

Locus	Variant	Frequency		Unconditional analysis[a]	
		Controls	Cases	OR (95%CI)	P value
HLA-DQB1	*04:01*	0.06	0.09	1.67 (1.35-2.06)	1.61E-06
	23(Leu)	0.06	0.09	1.67 (1.35-2.05)	1.73E-06
HLA-DRB1	*07:01*	0.05	0.09	1.63 (1.31-2.01)	1.12E-06
	104(Ala)	0.36	0.43	1.34 (1.2-1.5)	1.74E-07
	98(Glu)	0.36	0.43	1.34 (1.2-1.5)	1.74E-07
HLA_A (intronic)	rs2256919	0.44	0.38	0.75 (0.67-0.83)	2.21E-07

HLA, human leucocyte antigen; OR, odds ratio; 95%CI, confidence interval
[a]Obtained from multivariate unconditional logistic regression assuming an additive genetic model with sex, smoking status and principal components as covariates
The study-wide significant threshold was P=6 x 10^{-6} (Bonferroni correction)

Supplementary Table 9: Top associations of the HLA alleles with adenocarcinoma of Asian ancestry including a **smoking** as covariate on top of sex and principal components used in the ordinary analyses.

Locus	SNP2HLA vs HIBAG			
	European Oncoarray		Asian Oncoarray	
	Frequencies concordance [a]	Accuracy[b]	Frequencies concordance [a]	Accuracy[b]
HLA-A	0.99	0.91	0.99	0.98
HLA-B	0.99	0.98	0.95	0.93
HLA-C	0.99	0.97	0.99	0.97
HLA-DRB1	0.99	0.97	0.98	0.98
HLA-DQA1	0.86	0.88	0.85	0.84
HLA-DQB1	0.99	0.99	0.93	0.94
Average	0.97	0.95	0.95	0.94

a. Correlation coefficient between SNP2HLA and HIBAG imputed allele frequencies (rsq>0.7) in a random subset of 1000 samples from Asian or European Oncoarray series.

b. Accuracy calculation was used to assess overall imputation performance, defined as "the number of chromosomes with HLA alleles predicted correctly" over "the total number of chromosomes"; after QC (rsq>0.7).

Supplementary table 10: Comparison of imputed Oncoarray data using SNP2HLA with those HLA imputed genotypes obtained using HIBAG.

LOCUS	tagSNP ID (r^2)[a]	R2[b]
AH 8.1	rs3117582 (~0.81)	0.80
rs3117582	-	0.99
*HLA-B*0801*	rs2844531(~0.99)	0.98
*HLA-DQB1*06*	rs3135388 + rs62406300 (~0.84)	0.82
*HLA-DQB1*0602*	rs3135388(~0.99)	0.95
*HLA-DQB1*0603*	rs62406300 (~0.99)	0.97

AH 8.1, ancestral haplotype 8.1; HLA, human leucocyte antigen

[a] tag SNP ID and its concordance with the HLA variant between brackets.

[b] concordance between the OncoArray genotypes and the Affymetrix array for these variants in the 5,742 individuals where genotyping was available for both platforms

Supplementary table 11: Confirmation of the fidelity imputed genotyping of the OncoArray platform by considering concordance of these genotypes relative to genotypes obtained from analogous genotyping platform.

Locus	HLA variant	SCC European Ancestry [a]			SCC Asian Ancestry[b]		
		Frequency controls	Statistical Power probability (%)	INFO R2	Frequency controls	Statistical Power probability (%)	INFO R2
AH 8.1	*A1-B8-DR3-DQ2**	0.06	100	0.98	-	-	-
HLA-A	*01:01*	0.15	100	0.99	0.01	<80	0.96
HLA-C	*07:01*	0.15	100	0.99	0.002	<80	0.92
HLA-B	*08:01*	0.10	100	0.99	0.02	<80	0.92
	163(Thr)	0.28	100	0.99	0.16	100	0.84
HLA-DRB1	*03:01*	0.11	100	0.99	0.04	<80	0.97
HLA-DQA1	*05:01*	0.20	100	0.99	0.15	100	0.99
HLA-DQB1	*02:01*	0.11	100	0.99	0.09	96	0.95
HLA-DQB1	*06 global*[b]	0.24	100	0.99	0.22	97	0.9
	125 (Gly)	0.24	100	0.99	0.22	97	0.82

AH 8.1, ancestral haplotype 8.1; HLA, human leucocyte antigen; OR, odds ratio; 95%CI, confidence interval SCC, Squamous cell carcinoma

(R2) is the average across imputation batches

Statistical power calculations given European hits effects and the frequency found in controls of each ethnicity

[a] Calculations for the total SCC European samples: 4,581 cases and 15,439 controls

[b] Calculations for the total SCC Asian samples after metanalysis: 1,301 cases and 5,397 controls

*absent in Asians

Supplementary table 12: Imputation probabilities and statistical power calculations given the effects of European hits.

Locus	HLA variant	European Ancestry [a]			Asian Ancestry[b]		
		Frequency controls	Statistical Power probability (%)	INFO R2	Frequency controls	Statistical Power probability (%)	INFO R2
HLA-DQB1	*04:01**	0.0003	-	-	0.06	100	0.96
HLA-DRB1	*07:01*	0.13	100	0.99	0.09	100	0.99
	104(Ala)	0.29	100	0.99	0.36	100	0.99
HLA_A (intronic)	rs2256919	0.42	100	0.99	0.44	100	0.99

AH 8.1, ancestral haplotype 8.1; HLA, human leucocyte antigen; OR, odds ratio; 95%CI, confidence interval AD, Adenocarcinoma

(R2) is the average across imputation batches

Statistical power calculations given European hits effects and the frequency found in controls of each ethnicity

[a] Calculations for the total AD European samples: 7,088 cases and 15,439 controls

[b] Calculations for the total AD Asian samples after metanalysis: 4,576 cases and 5,129 controls

* almost absent in Europeans

Supplementary table 13: Imputation probabilities and statistical power calculations given the effects of Asian hits and imputation probabilities.

Locus	Variant	Frequency		Unconditional analysis[a]	
		Controls	Cases	OR (95%CI)	P value
Haplotype HLA class II[b]	DRB1*13:01-DQA1*01:03-DQB1*06:03	0.07	0.06	0.85 (0.77-0.94)	7.50×10^{-04}
HLA-DQA1	*01:03*	0.08	0.07	0.84 (0.76-0.92)	1.79×10^{-04}
HLA-DQB1	*06:03*	0.07	0.06	0.84 (0.76-0.93)	5.06×10^{-04}
HLA-DRB1	13:01	0.07	0.06	0.85 (0.77-0.93)	9.27×10^{-04}

HLA, human leucocyte antigen; OR, odds ratio; 95%CI, confidence interval

[a]Obtained from multivariate unconditional logistic regression assuming an additive genetic model with sex and principal components as covariates

[b] Haplotype associated associated with head and neck[14] and cervical cancer[15], both squamous cell carcinomas linked to HPV infection

The study-wide significant threshold was P=6.03 x 10^{-6} (Bonferroni correction)

Supplementary Table 14: Association of the HLA-DRB1*1301–HLA-DQA1*0103–HLA-DQB1*0603 head and neck and cervival cancer haplotype with squamous cell carcinoma of European ancestry.

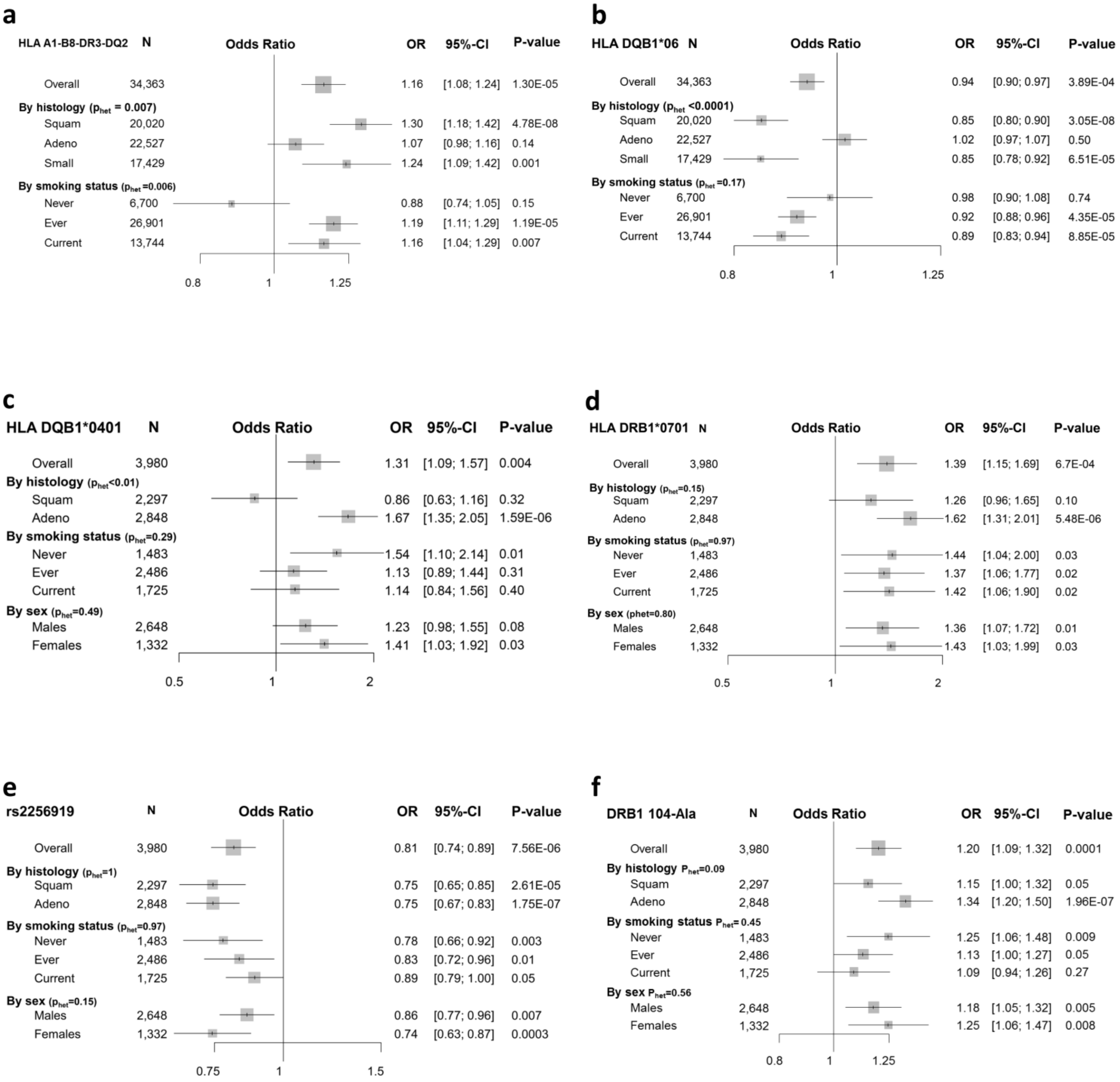

Supplementary Figure 1: Stratified analyses of top-ranking variants for all lung cancer subtypes of European (**a-b**) and Asian ancestry (Oncoarray) (**c-f**). Results are shown for (**a**) the AH8.1 haplotype (*A1-B8-DR3-DQ2*), (**b**) *HLA-DQB1*06*, (**c**) *HLA-DQB1*0401*, (**d**) *HLA-DRB1*0701*, (**e**) rs2256919, (**f**) amino acid 104-Ala in HLA-DRB1. Stratified analyses by histology and smoking status were obtained from multivariate unconditional logistic regression assuming an additive genetic model with sex and principal components as covariates; stratified analyses by sex included just principal components as covariates. N: total number of cases and controls. The study-wide significant threshold is $P = 6 \times 10^{-6}$.

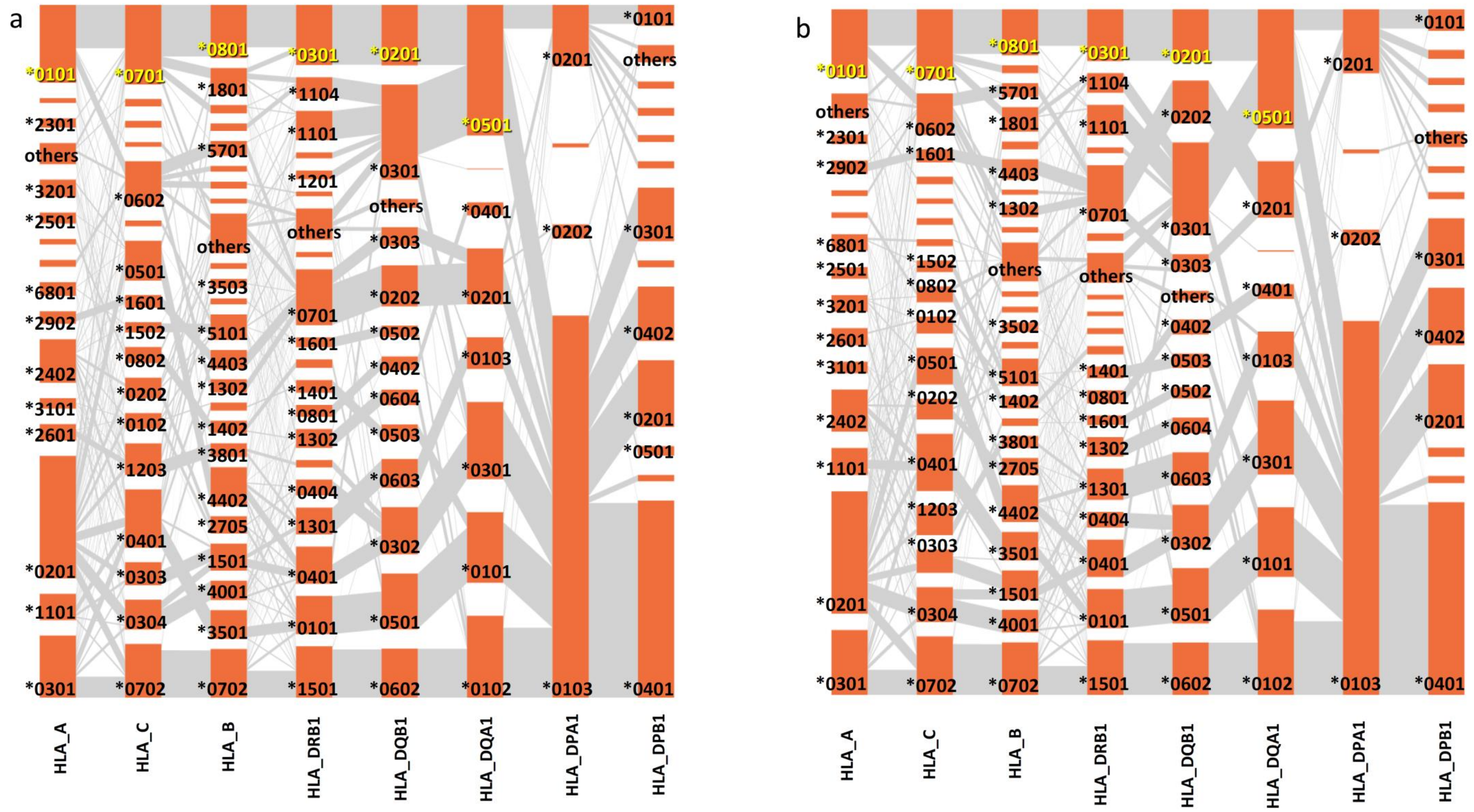

Supplementary Figure 2: Haplotype structure of HLA alleles for lung cancer cases (a) and controls (b) of European ancestry. The vertical stacked bar indicates each of the 8 HLA genes, and the queues of the bars correspond to their physical order in the MHC region. A tile of a bar indicates an HLA allele, and a segment connects 2 alleles on adjacent genes. The height of the tile and the thickness of the segment correspond to the allele frequency of the HLA allele and haplotype frequency between the 2 HLA alleles, respectively. The existence of the common haplotype AH8.1 (yellow) is clearly shown, whose frequency is increased in cases regarding controls.

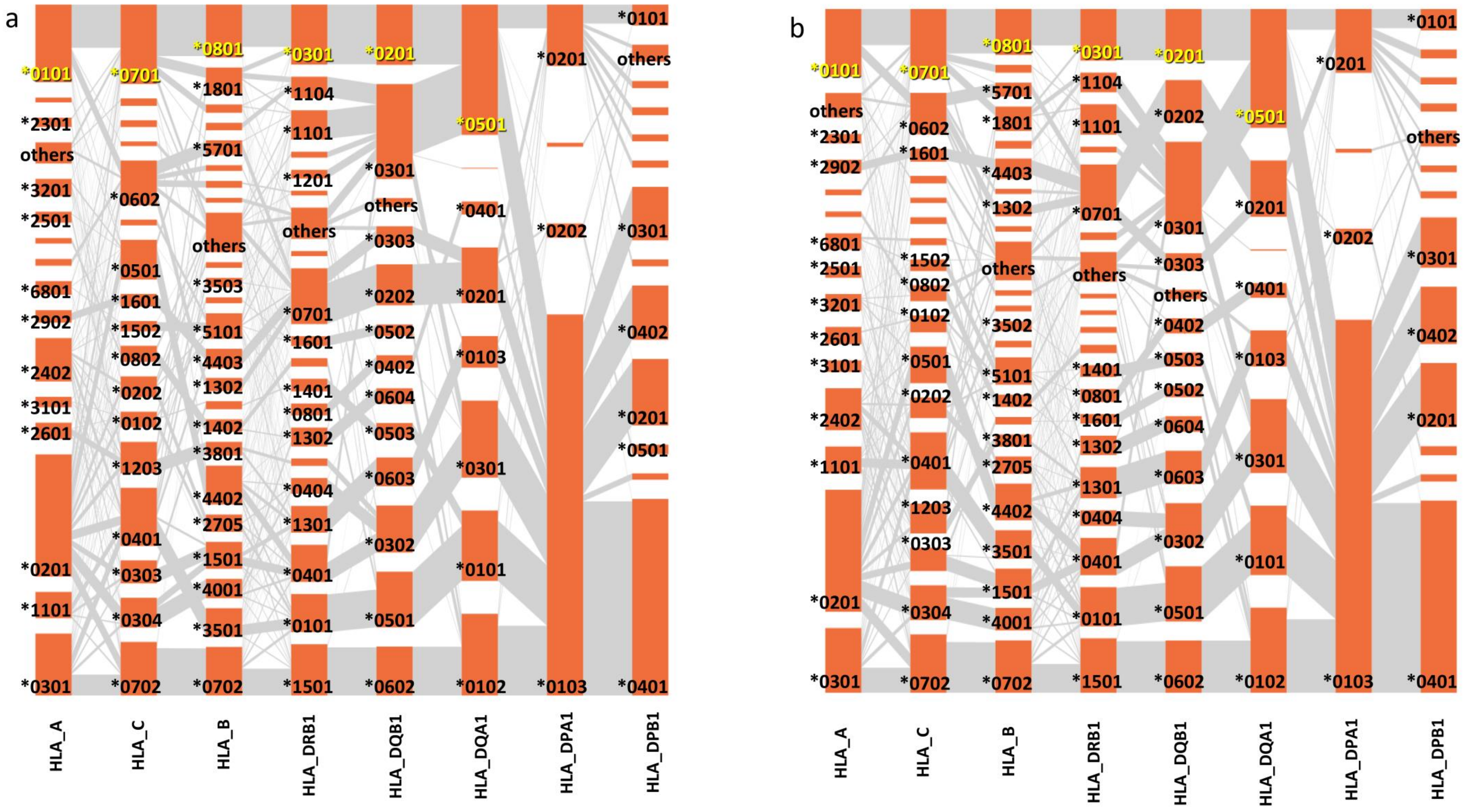

Supplementary Figure 3: Haplotype structure of HLA alleles for lung cancer cases (a) and controls (b) of Asian ancestry. The vertical stacked bar indicates each of the 8 HLA genes, and the queues of the bars correspond to their physical order in the MHC region. A tile of a bar indicates an HLA allele, and a segment connects 2 alleles on adjacent genes. The height of the tile and the thickness of the segment correspond to the allele frequency of the HLA allele and haplotype frequency between the 2 HLA alleles, respectively. Independent associated alleles with lung adenocarcinoma are shown in blue and yellow.

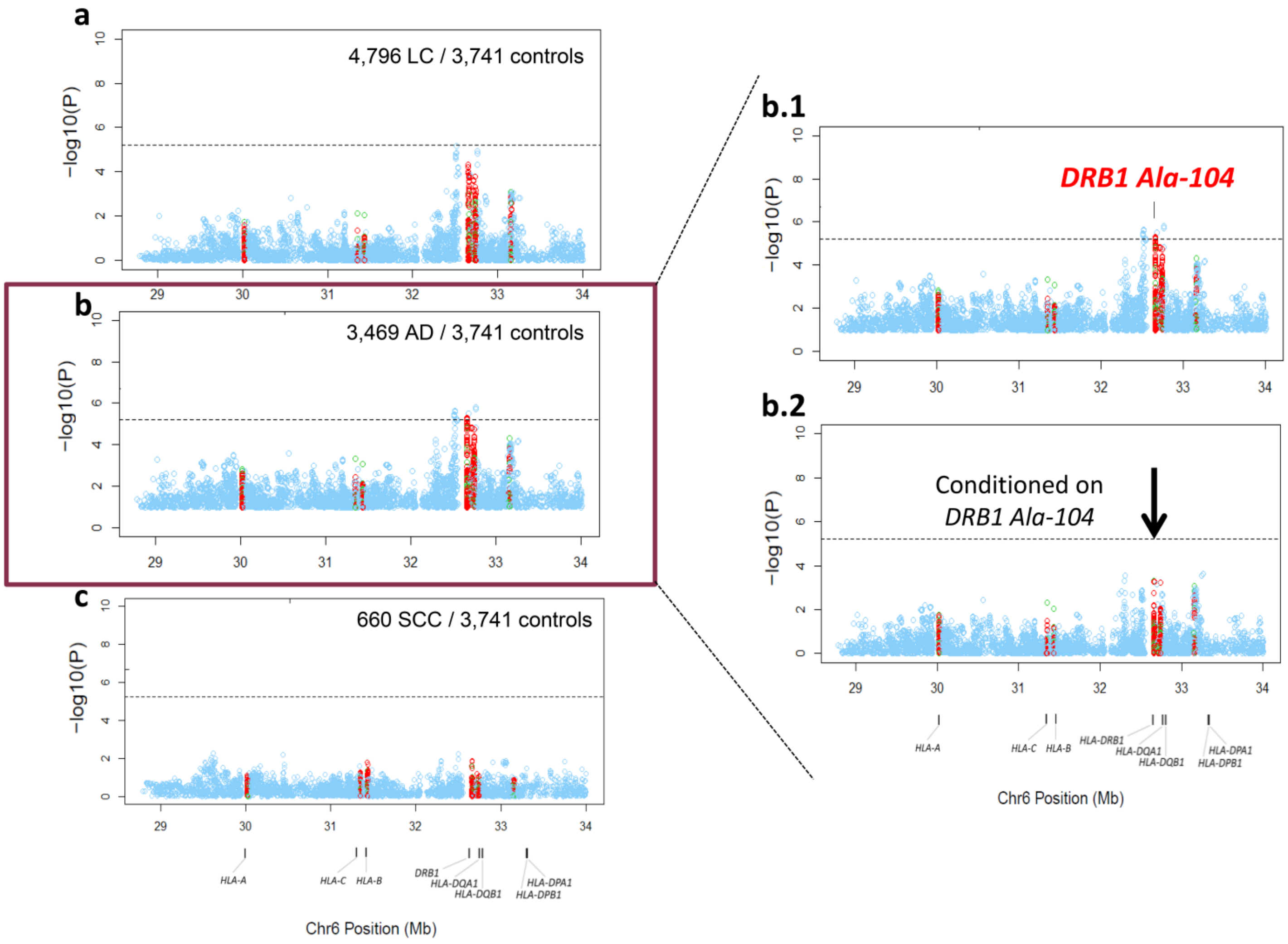

Supplementary Figure 4: Replication Asian regional association plots of variants in the MHC region and lung cancer overall and major histologies (a-c); **plots of stepwise conditional analyses for adenocarcinoma (b.1-b.2).** Each panel on the left shows the association plot for each unconditioned analysis **(a)** lung cancer overall, **(b)** adenocarcinoma, **(c)** squamous cell carcinoma. The association for each locus used for conditioning is shown in green in each panel **(b.1)** unconditioned, **(b.2)** conditioned on amino acid Ala-104 in *HLA-DRB1* sequence. Circles represent –log10 (*P* values) for each binary marker using the imputed allelic dosage (between 0 and 2). The dashed black horizontal lines represent the study-wide significant threshold of $P= 6 \times 10^{-6}$. The physical positions of HLA genes on chromosome 6 are shown at the bottom. The color of the circles indicates the type of marker; light blue – SNPs, green - classical HLA alleles and red - amino acid polymorphisms of the HLA genes.

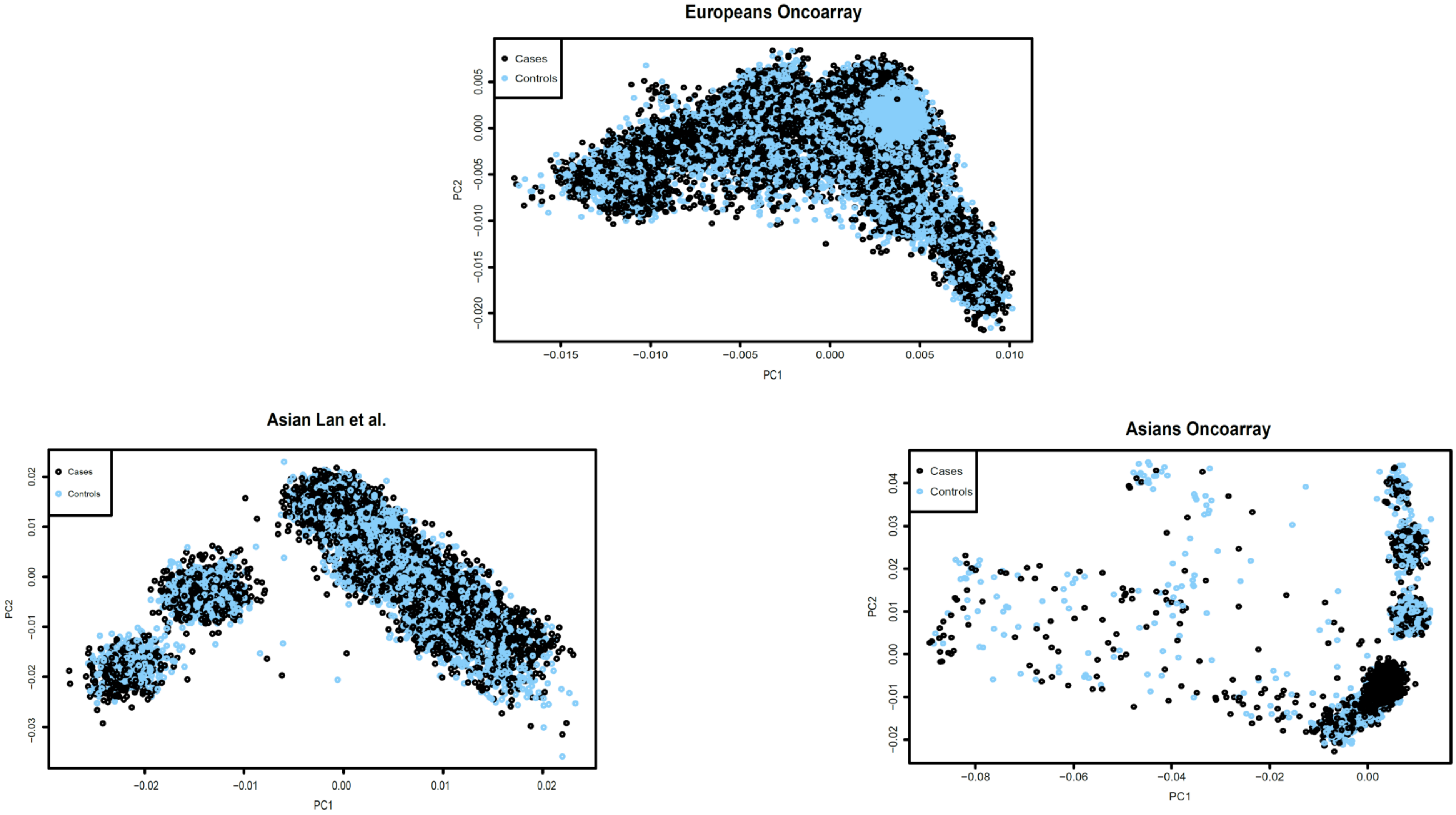

Supplementary Figure 5: Principal-components analysis plots. Plots are shown for all study participants for Europeans (top), Asians from Oncoarray (bottom right) and Asians from Lan et al. (bottom left). Principal component 1 is displayed on the *x* axis, and principal component 2 is displayed on the *y* axis. Blue dots are controls, and black dots are cases.
